# Party activism: the permeability of the asylum protest arena in Austria

**DOI:** 10.1080/14742837.2019.1567321

**Published:** 2019-01-29

**Authors:** Leila Hadj Abdou, Sieglinde Rosenberger

**Affiliations:** a Migration Policy Centre, Robert Schuman Centre for Advanced Studies, European University Institute, Florence, Italy; b Department of Political Science, University of Vienna, Vienna, Austria

**Keywords:** Protest, immigration, asylum, Austria, political parties, deportation

## Abstract

This article contributes to the growing field of studies on party–protest linkages that highlight the dynamic nature, complementarity and fuzziness of the parliamentary arena and protest arena. Taking the policy field of asylum, it investigates, first, the conditions for the permeability of the protest arena for party activism and, second, the ways in which party activism shapes and transforms the protest arena. The empirical observations refer to Austria, which has a political framework with highly politicized immigration, strong political parties and a weak protest culture. Methodologically, the paper combines a protest event analysis with two in–depth case studies on protests. The authors argue that the openness of the asylum protest arena for parties is characterized by modest protest demands, and depends on the dominant political position as well as the decision making structure regarding the protest issue. The article demonstrates that pro-asylum protests are less open to political parties than anti-asylum protests, which are in tune with the dominant political position on asylum in Austria. The findings also show that anti–asylum protests are not only more likely to attract the involvement of political parties, but also tend to become instrumentalized for party–competitive ends. Pro–asylum protests, in contrast, keep their substantive, grievance–focused orientation even when political parties step in.

In the light of political distrust and dissatisfaction with elites, protest politics is of growing relevance in many representative democracies (Krastev, 2014). Social movements and protest groups communicate demands that are silenced or unaddressed by the authorities who hold the decision–making power and voice grievances with the aim of influencing attitudes and decisions. Increasingly, political parties and state representatives also turn to the streets, develop ties with protest networks or even initiate extra–parliamentary protests. These developments highlight that the boundaries between institutionalized and non–institutionalized politics, between protest groups and state actors are ‘fuzzy and permeable’ (Goldstone, 2003, p. 2).

Scholarship on political parties and social movements has provided highly relevant insights into the alliances and linkages between the parliamentary and protest arenas (e.g. Della Porta, Fernandez, Kouki, & Mosca, 2017; McAdam & Tarrow, 2010). Scholars have identified different forms and trajectories of the interaction between parties and movements. Movements ‘compete with parties, movements infiltrate parties, […] parties spin off movements […], movements appear within parties […]’ (Garner & Zald, 1987, cit. after Semenov, Lobanova, & Zavadskaya, 2016, p. 83). Empirical research on the specific conditions for the permeability of the protest arena for party activism, however, is still scarce. Nor is much known about whether and how party activism shapes and transforms the protest arena. This article is interested in filling this lacuna by providing insights on the conditions that facilitate the openness of the protest arena for party actors and the impact that party activism has on key features of the protest arena. By ‘party activism’ we refer to mobilization activities by parties beyond the representative channels.

To observe the openness of the protest arena for party activism, we take up the contested issue of asylum (Van der Brug, D’Amato, Berkhout, & Ruedin, 2015). We investigate protests in favour of asylum seekers staying in the country and protests against accommodation for asylum seekers.

Two research strands guide the analysis: First, protest scholarship that emphasizes endogenous protest features like forms and goals of protests (Probst & Bader, 2018); and second, the political opportunity structure approach that accounts for the environment where protest politics takes place (Rucht, 1988). By using these approaches, we expect that the permeable protest arena will be best understood through exogenous political opportunities plus characteristics that are endogenous to the protest arena.

The study refers to Austria’s political system, a national context characterized by a high level of party dominated political culture, a strong negative politicization of the issues of immigration and asylum, and a weak protest culture from below (Merhaut & Stern, 2018). As we show, this context makes anti–asylum protests much more permeable to party involvement than pro–asylum protests. The concrete findings we discuss in this article are thus specific to the Austrian context, but, at the same time, our analysis allows for generalizable insights. In more general terms, we argue that party activism in the extra-parliamentary arena depends on specific features of the protest, such as the political orientation of protests. The political orientation of protest matters, given that the overall dominant political position on the issue shapes the permeability for party activism. Moreover, the scope of protest demands is another facet that affects permeability. Party activism is mostly confined to engagement in protests with modest demands. Our analysis also highlights the necessity of taking the decision-making structure regarding the protest issue into account in order to understand party activism. The more political responsibility (for a certain protest issue) party actors have, the less active they will be in the protest arena.

The article also provides insights on how the involvement of parties in protest activities shapes the mode of doing extra–parliamentary politics. We demonstrate that political parties involved in pro–asylum protests respond to citizens’ concerns and supply additional resources to protest groups without changing the bottom–up character of the protest. Party activism in anti–asylum protests, on the contrary, tends to transform the protest arena, turning it into a space of competitive party politics.

In the following section, we introduce the theoretical and analytical framework on which the article is based. We then provide information on the national context of Austria and on the data and methods before proceeding to our findings.

## Theoretical & analytical framework

Protest and protest arena form the key concepts in this article. To clarify what we mean by protest, we follow Opp’s definition (2009, p. 44): a political protest is the ‘joint (i.e. collective) action of individuals aimed at reaching their goal(s) by influencing decisions of a target’. Protest actors aim to challenge and shape the political decisions made in the parliamentary arena. The notion of the protest arena refers to a site of political mobilization outside parliamentarian channels. The protest arena differs from the parliamentary arena in its modal forms of engagement (Hutter, 2013). While the key form of engagement in the protest arena is non–institutionalized, challenging elites and covering a range of repertoires, from public statements and petitions to demonstrations, strikes and riots, in the parliamentary arena engagement is institutionalized; it is about voting by citizens and decision–making through legislative channels by political parties and governmental actors. Social movement and protest studies have attributed distinct actor groups to each arena (Tilly, 1978). The parliamentary arena has been understood as comprising political parties and administrative officials, as opposed to the protest arena, which is made up of civic groups, individual citizens and grassroots initiatives that challenge the existing political authorities and institutions. Today it is widely acknowledged that the ‘two worlds of inside (institutionalized, conventional) and outside (protest, unconventional) politics are not as neatly separated’ (Kriesi, 2015, p. 668). In this vein, studies have begun to pay attention to players who ‘shift arenas in the search for new advantages’ (Jasper, 2015, p. 10). Scholarly interest has focused on the strategic underpinnings of alliances and coalitions between institutional and non–institutional actors (e.g. Van Dyke & McCammon, 2010).

In this article we focus on political parties, who are the key actors in the parliamentary arena. In the domain of migration and asylum, challenger parties, i.e. parties that have been largely untarnished by office (Hobolt & Tilley, 2016), are known to be influential actors in politicization. Challenger parties on the left and on the right mobilize voters upon a new transnational cleavage, which is driven by increased immigration and exacerbated by cultural, religious and economic insecurity (Hooghe & Marks, 2018, p. 110). The reconfiguration of political conflicts (Hooghe & Marks, 2018) has provided new opportunities for reshaping and tightening relations between parties and protest groups (Hutter, Kriesi, & Lorenzini, 2018). Parties join protest groups or initiate activities to widen their scope for mobilizing citizens and putting pressure on the authorities (Rucht, 2018).

In turn, for mainstream parties, i.e. parties that frequently alternate between government and opposition (Hobolt & Tilley, 2016), immigration and asylum are policy issues characterized by conflicted positions. The migration and asylum questions cut across traditional cleavages like class and religion. Mainstream parties who emerged upon these traditional cleavages are torn on these issues, while also being pushed by challenger parties who mobilize heavily around these issues (Odmalm & Bale, 2015). Notwithstanding their conflicted positions, mainstream parties, and even parties holding office (Verhoeven & Duyvendak, 2017), engage in the protest arena.

Very often, to understand the emergence and dynamics of protests, the scholarship has turned to political opportunity structure approaches. Political opportunities include stable contextual features like the institutional framework, as well as dynamics like shifts in power relations (Rucht, 1988, p. 310; Caiani & Borri, 2016, p. 74). For the issue dealt with in this paper, we draw on the political opportunity perspective and combine it with an approach that looks at conditions that are endogenous to the protest arena. Against this background, three expectations guide this analysis, as follows.

First, some party actors are office holders, responsible for decision making, while others are not. We regard the place of the party actors in the decision–making structure on the protest issue to be a relevant condition for party activism. Adapting the idea of the tension between political parties’ responsibility and responsivity when siding with protest groups (Hutter Kriesi, & Lorenzini, 2018), we thus argue that the greater the political responsibility of party actors in regard to a certain contentious issue, the less active they can be expected to be in this protest arena.

Second, we also expect the dominant political position on the specific protest issue to be a relevant condition for party activism. Drawing on the literature on the politicization of migration and asylum (Van der Brug, D’Amato, Berkhout & Ruedin, 2015), we see the strength of the actors politicizing the issue of asylum in the parliamentary arena, as well as the attitudes among the wider citizenry on the issues of asylum and immigration as relevant indicators for the dominant political position in the field of asylum.

Finally, a third guiding expectation is based upon insights coming out of research that focus on factors that are endogenous to the protest arena, in particular the demands raised. For instance, Probst and Bader (2018), in their study of a rather unusual case, namely the involvement of right–wing politicians in pro–migration protest activities, show that one of the main conditions that allowed these strange bedfellows to emerge was the personifying character of the protest demands. Protest, which focuses on the grievances of particular migrants and does not challenge the restrictive migration regime as such, the findings of these authors suggest, is susceptible to party activism, even among parties with conflicted or contradictory migration agendas. We suspect, therefore, that the scope of demands will matter for party activism and the permeability of the protest arena.

## The political context of Austria, data & methods

The political context of Austria in which we investigate the permeability of the protest arena is characterized by three relevant features. First, political parties enjoy great relevance. According to Van Biezen and Kopecký (2014, p. 178), Austria is still one of the strongholds of Europe in terms of party patronage, even if there has been a decline in recent decades, along with the rise of challenger parties, the Green Party and the Freedom Party (FPÖ). The mainstream parties are the Social Democratic Party (SPÖ) and the People’s Party (ÖVP), both of which are long–term governmental parties. The Green Party grew from an ecological movement into a political party. The FPÖ evolved from a nationalistic fringe party into an electorally successful populist, radical right–wing party.

Second, Austria is characterized by low intensity in terms of extra–institutional activities. Political demands are primarily expressed within the institutional channels (Dolezal & Hutter, 2007). With its consensual decision–making institutions, Austria has enjoyed an inclusive mode of politics. However, this inclusiveness primarily covers the actors within the social partnership, whereas social movements have usually been marginalized, with the exception of the environmental movement. In the 1980s, the Green Party emerged from the ecological movement.

Finally, immigration and asylum are highly politicized issues, which is mirrored in public attitudes. Public opinion towards immigration, including asylum seeking immigration, has been relatively hostile and a high proportion of the population is in favour of restrictive policies (Heath & Richards, 2016). Immigration started to become a major issue in the early 1990s and has remained so ever since. Extra–institutional actors have played quite a marginal role in this process. The politicization of the issue is strongly related to the FPÖ and to a lesser extent the Green Party. For both parties, the issue of migration has contributed significantly to their electoral growth (Gruber, 2014, p. 133; Meyer & Rosenberger, 2015, p. 48). Most notably, the FPÖ is one of the strongest populist radical right–wing parties in Europe today, having gained 26 per cent of the vote in the 2017 general elections. As in other European countries (Odmalm & Bale, 2015), the mainstream parties hold mixed positions on the issue of migration in an attempt to bridge conflicting preferences among their electorates.

We take up the asylum issue to study party–protest linkages for the following reason: asylum is an issue that is not only politicized by political parties but is also of concern for extra-parliamentary protest groups, ordinary citizens and advocacy organizations. Hence, the asylum protest arena studied includes protest instances against the deportation of rejected asylum seekers and protest events against accommodation facilities for asylum seekers. These two issues have been the major topics of the asylum protests in Austria. As the period of investigation, we chose 2004 to 2016 for two reasons: first, the rate of forced removal of rejected asylum seekers started to rise in and after 2004; second, in 2004 the Basic Welfare Support Agreement was introduced, which regulates the reception of asylum seekers based on the EC Reception Directive (2013/33/EU). The end date of 2016 was chosen to include mobilization before and during the large-scale asylum migration of 2015.

As regards the methods for the data collection and analysis, we combined a protest event analysis (PEA) of individual actions (Koopmans & Rucht, 2002) with two in–depth qualitative cases. The PEA is based on media reports collected from the two quality papers, Die Presse and Der Standard, which we accessed through the Nexis database. Die Presse has a conservative orientation and Der Standard is considered liberal. We used both newspapers in a complementary way to account for and reduce any ideological selection bias. This is not to say that we could control for the overall selection bias of these newspapers. Although media content analysis has become an established method in social movement research, the picture the media draw is a highly selective reality (Koopmans & Rucht, 2002, p. 252). There is a trade–off between analysing protest events with public relevance and an accurate representation of de facto activism, which might be significantly higher than that reported in media outlets. Based on keyword research, we identified a total of 252 reported protest events (PE) pertaining to resistance to the deportation of rejected asylum seekers, and 65 protest events concerning resistance to refugee facilities.^1^ All instances were manually coded. We only coded protest events if the form was reported by the media and we excluded unspecific reports that simply mentioned protest. For protest events with party involvement, we coded the specific party actor, their role (distinguishing between initiating and supporting), their repertoires (e.g. petitions or demonstrations), the main demand of the protest event (e.g. to stop the deportation of a specific individual, to stop running a refugee facility) and the territorial level (national, regional, local).

The PEA was supplemented by tracing two key cases of protest to obtain greater knowledge on party–protest relations. The cases selected qualify as key protest cases, with high protest activity including a high level of party activism. These cases allowed us to identify not only how parties get involved, but also how parties impact on the characteristics of the protest arena itself. The analysis here is based on a collection of news reports and protest material, as well as secondary literature.

## Permeable asylum protest arena: findings from the protest event analysis

In this sub-chapter, we present the findings based on the PEA, identifying the degree of party activism and two relevant conditions that facilitate the permeability of the asylum protest arena, namely modest scopes of demands and local grievances within a national governance structure.

### Degree of party activism

Our data show that the number of protest events (PE) differs between pro – and anti–asylum protest activities. Protests against asylum facilities are, overall, less frequent than protests against the deportation of rejected asylum seekers. In the two national media outlets, we detected 252 protest events related to the issue of anti–deportation, but only 65 events related to accommodation facilities for asylum seekers. In terms of party involvement, interestingly, we found 39 incidents of party involvement in anti–asylum protests, i.e. party activism, in over half of the protest events. For protests against the deportation of rejected asylum seekers, we found party–political participation in only 37 events, or 15 per cent of the events. Protest activities in favour of asylum seekers being allowed to stay are thus more frequent but with less party involvement, compared to protests against facilities for asylum seekers. Political parties are predominantly engaged in anti–asylum protests (see Table 1).

**Table 1.** Overview of party activism in the asylum protest arena.

Protest events (PE)	‘Pro-asylum’ protests	‘Anti-asylum’ protests
PE (TOTAL)	252	65
PE with party activism	37	39
FPÖ	0	18
Greens	16	1
SPÖ	12	13
ÖVP	16	9
Other parties	4	1
TOTAL PE (pro & anti asylum)		317
TOTAL PE with party involvement		76 (24 per cent)

Total N deportation protests (‘pro-asylum’) = 252; total N accommodation protests (‘anti-asylum’) = 65.

For ‘anti–asylum’ protests, we see a peak of activism, including party activism, in 2015 (24 PE, eight of which involved party activism), and a constant level of party activism in 2016 (eight PE), despite the decreasing overall protest activity (12 PE). Prior to these years, ‘anti–asylum’ protests happened in very low numbers, which highlights the relevance of the 2015 refugee movements for activism. Pro–asylum activism peaked in the years 2007 (45 PE) and 2010 (49 PE) in relation to prominent cases of individual deportation, and party activism correspondingly peaked in 2007 (13 PE with party activism).

Differentiating between parties, both challenger and mainstream parties engage in the protest arena. The challenger and anti–migration party FPÖ engages exclusively in anti–asylum protests. It not only aligns itself with protest actors, but also initiates events (the FPÖ engaged in 13 out of the 18 protest events). The FPÖ is known to have persistent contacts with far–right movements (Bailer-Galanda & Neugebauer, n.y.). The Green Party, in contrast, participates almost exclusively in pro–migration protest activities. It evolved from a social movement into a party (Dolezal, 2010, p. 539) and can thus be assumed to have preserved something of a movement culture. The mainstream parties (SPÖ and ÖVP) are also active. The SPÖ, and to a lesser degree the ÖVP, participate in anti–asylum protest action. Both have initiated as well as supported protests against the accommodation of asylum seekers, and both parties have acted as supporters in pro–asylum protests. The SPÖ initiated seven of the 13 PE in which it was involved, and the ÖVP initiated two out of nine PE. In contrast to challenger parties, however, they engage in both pro- and anti-asylum protests, which mirrors their conflicted position on immigration, while challenger parties have a clear–cut position on the matter.

The fact that even in a context of a low protest culture parties engage in the protest arena reaffirms the scholarship that emphasizes the fuzziness of the boundaries between the streets and parliament. It also mirrors the strong position of political parties in Austria as opposed to social movements. The finding corresponds with other recent studies. An analysis of longitudinal data on anti–deportation protests in Austria, Germany and Switzerland found that roughly 10 per cent of all actors involved are representatives of political parties and governmental authorities (Ruedin, Rosenberger, & Merhaut, 2018). For anti–asylum protests, Haselbacher and Rosenberger (2018) investigated resistance to the establishment of accommodation centres between 2014 and 2017 and found that institutional actors, especially mayors, were the main actors involved in this kind of protest. In contrast, citizens are less present and their activism is reactive rather than proactive; they push politicians but initiate protest events only to a very limited extent (ibid.).

### Modest implementation demands

For protests in favour of asylum seekers, we differentiate between two types of protest demands: First, moderate claims concerning the implementation of deportation policies in individual cases; and second, radical claims that challenge these policies or the underlying political order. Moderate implementation claims, in contrast to radical claims, demand an ‘exemption from the rule’ rather than questioning the rule and its underlying order as such (cf. Probst & Bader, 2018). For the pro–asylum protest arena analysed, the most common demands are for a halt to specific deportation orders. We counted 143 major claims against the deportation of a specific individual, out of a total of 252 made during the selected period (see also Ruedin, Rosenberger & Mehrhaut, 2018). Party activism in anti–deportation protests does not deviate from this overall pattern of moderate claim making. Party political actors mostly advocated for a halt to a specific deportation order (20 out of 37 major claims identified).

For anti–asylum protests, the claims made relate to the rejection or a proposed reform of an existing asylum accommodation facility in a particular locality. Similar to the pro–asylum arena, these protest demands refer primarily to the implementation of decisions rather than the broader policy regime on refugee protection. We thus categorized them as moderate implementation claims. In addition, we identified claims against the existence of asylum seekers, which we categorized as radical. These claims manifest themselves in violent protests, such as attacks. Twenty–one out of the 65 PE contained a radical claim. For party actors, claim making is focused on implementation, with the majority of claims (20) being made against the establishment of a new facility or the removal of an existing facility (18), and a minority of claims (five) demanding the reform of an existing facility.

We conclude that the asylum protest arena, which is open to and characterized by party activism, is typified by modest protest demands focused on the implementation of decisions rather than on challenging the broader asylum regime.

### Local grievances and national responsibility

The PEA data demonstrate that the asylum protest arena in which political parties engage is mostly subnational. Both anti – and pro–asylum activism take place at the subnational level and party activism in particular hardly ever spreads to the national level. For anti–asylum protests, this subnational pattern has to do with the locality of the grievance – the erection or the presence of an accommodation centre in the immediate neighbourhood. In the pro–asylum arena, grievances often arise when a deportation order has been issued for an individual with whom activists have developed social ties (see also Rosenberger & Winkler, 2014). In particular, politicians affiliated with the ÖVP – which for more than ten years has supplied the Minister of the Interior in charge of migration and asylum matters – are most active at a local level. The SPÖ, the FPÖ and the Green Party are active at the local and provincial levels.

The vertical nature of the decision–making power explains this subnational pattern. In Austria the administrative responsibility for the accommodation of asylum seekers is shared between the federal government and the provinces. The federal government is responsible for asylum legislation, procedures and initial reception. The provinces are obliged to accommodate asylum seekers, i.e. to provide, supervise and manage reception facilities (Rosenberger & König, 2011). Regarding the forced removal of rejected asylum seekers, the federal government is responsible for issuing and implementing deportation orders. Against this institutional background, the protests challenge the authorities in the upper political tiers who are in charge of decision making. This vertical power structure allows subnational politicians – not only from the challenger parties, but also those affiliated with governmental parties – to express their solidarity with the concerns of local protest groups. In particular, mainstream parties tend to limit their unconventional activities to local issues and focus on implementation rather than legislative claims. In sum, this subnational protest pattern can be viewed as translating the tension between responsibility and responsivity (Hutter, Kriesi & Lorenzini, 2018) into party activism. Parties without direct responsibility enjoy greater leverage in terms of being active in the protest arena, while the party actors in charge are more constrained (cf. ibid.); they have to take into account various, partly contradictory, interests and obligations, which include refugee protection legislation as well as demands by voters, who are tough on immigration.

## Asymmetric permeability: findings from the case studies

To further substantiate our insights into the facilitating conditions and address the question of how party activism transforms the protest arena, we trace two key protest cases. In terms of the political context, it should be mentioned that they occurred in the same national political context of a coalition government between the SPÖ und ÖVP; and both protests exhibited intense party activism across the political spectrum.

### Pro–asylum party activism

The selected protest case lasted from 2007 to 2010 and centred on preventing the deportation of a family who sought asylum in Austria in the aftermath of the Kosovo crisis. After the asylum request was rejected, the family used all legal recourse available, but without success. Just before the deportations were due to be executed, the daughter went into hiding and posted a video in which she threatened to commit suicide unless the removal orders were withdrawn.

The triggers for the large subsequent protest were close social relations, empathy and a moral shock over the threat of forced removal. Initiated by local acquaintances of the family, the claim was to prevent the deportation of this family. The main addressee was the Federal Ministry of the Interior responsible for issuing the deportation order. From the very beginning, the protest groups enjoyed the backing of the local community and local politicians. The protest eventually went national and was joined and amplified by political parties from all political levels. A study conducted by Gruber, Herczeg, and Wallner (2008, p. 117), which analysed newspaper and television reports around the case, concluded that politicians were the key actors; civil society was less vocal and the potential deportees were marginalized voices.

The wide scope of the protest – which is unusual for Austria – was linked to the favourable public perception of the family and their depiction as particularly deserving because they were viewed as having integrated into society. As a result of the frame of deservingness in the public debate, the family initially received significant support from the municipality where they lived, the district authorities and the provincial government, which filed a humanitarian residence permit request for the family. This supportive behaviour drastically changed after some crime–related accusations linked to the family surfaced in the media in late 2007. Following this, politicians, including subnational ones like the mayor, refrained from supporting the family further (Eybl, 2009, p. 96).

In the course of the protest, the modest implementation claim to prevent the family’s deportation turned into a more radical claim for new legislation. In 2010, some 20,000 people took to the streets to challenge the authorities on the deportation order and called for a ‘human asylum policy’ (‘Genug ist genug’, Die Presse, 1 July 2010). This demonstration, held under the slogan ‘Enough is Enough’, was one of the rare mass protests that has happened in Austria in the field of migration and asylum.

The initial protest network, which consisted of friends, students, workmates and the local priest, was sceptical about party involvement. Media material and interviews with local citizens (Eybl, 2009, p. 93) provide evidence that these activists did not appreciate an explicit party political tag being placed on their engagement and expressed fears that the protests would be monopolized by political parties. The activists wanted to keep their protests ‘pure’ and free from political parties so that people with diverse and non–political affiliations would be encouraged to join.

Party activism occurred nevertheless. Overall, the Green Party was the most active and undertook a variety of initiatives to support the family’s right to stay. Most notably, an email petition was launched in 2007, which was directed at the federal government. High–ranking Green party officials took part in demonstrations at the local, regional and national levels. Extra–institutional activities were combined with parliamentary arena tools. The Greens called for a special session in parliament to table a motion of no confidence against the Minister of the Interior and demanded the introduction of a right to stay and an immediate halt to the deportation of ‘well integrated’ families. The party leader also wrote an open letter to all provincial governors asking them to support the motion for a right to stay; and several talks with members of government were sought by regional and national party representatives to prevent the deportation.

Initially, when the municipality stood by the family, the municipal council filed a unanimous decision not to deport the family and the local FPÖ branch also voted in favour of this (symbolic) decision. The FPÖ did not partake in any extra–institutional protests surrounding the case, but they dealt with the case extensively in the parliamentary arena. In response to the Green Party’s involvement, the FPÖ repeatedly called for the prosecution of Green representatives (Eybl, 2009, p. 90). In 2009 the FPÖ held its final EU–parliamentary election campaign event in the village where the family lived.

The two mainstream parties, who were in national governmental office during the time of the protests, played an ambiguous role. Politicians from the SPÖ and the ÖVP were most likely to advocate for the family at subnational levels. Some national, high–ranking politicians of the ruling SPÖ also joined the activists in campaigning against the deportation of the family. National ÖVP representatives did not show any support for the family, given that the Ministry of the Interior was held by the ÖVP. The position of both of the SPÖ prime ministers in office during the protest period was characterized by inertia or they sided with their conservative coalition partner.

### Anti–asylum party activism

In 2009 the Minister of the Interior (ÖVP) formed an agreement with the mayor (ÖVP) of the small village of Eberau in the province of Burgenland to create an initial reception centre for 300 refugees there. The public announcement of the plan was followed by intense local protests. Within a few days, a citizens’ initiative had formed to mobilize against the centre and demand the resignation of the mayor. Soon after that, the mayor apologized for his previous position in support of the facility. The entire municipal council eventually signed a petition organized by the initiative to prevent the construction of the centre (‘Eberau’, Die Presse, 1 January 2010). The protest was successful – the federal proposal for the accommodation centre was withdrawn.

The case quickly became immersed in party politics, and soon the conflict spread beyond the local level. The FPÖ initiated a protest event in the village and the national party leader gave an inflammatory speech in front of 800 residents (‘Zelte aufschlagen’, Kronen Zeitung, 16 January 2010), equivalent to nearly the entire population of the village. On that occasion the party declared that, should the construction go ahead after the referendums, it would return and put up a protest camp. In turn, the Greens supported an online platform of independent activists collecting signatures against the referendums (Weisgram, 2010). The regional SPÖ governor used the discontent of the local citizens to attack the ÖVP Minister of the Interior. He collected signatures against the centre, called a referendum on the issue in the village and insisted on an additional regional referendum. In the local referendum 90.14 per cent of those who voted were against the centre and in the following provincial referendum 94.5 per cent of voters expressed their opposition to the establishment of such a centre in their province (Prior, 2010). The (SPÖ) prime minister backed the calls for a referendum. After the emergence of the first waves of protest, regional ÖVP officials also turned against the Minister of the Interior. Challenging the federal government in this case was especially beneficial for the regional SPÖ party organization, governing the province. It was part of a successful strategy to mobilize voters for the upcoming 2010 provincial election, which the SPÖ won again. Thirty-eight per cent of the surveyed electorate stated that the reaction against the facility and the direct democratic instruments applied by the SPÖ were decisive factors in casting their vote for the party (Filzmaier, Perlot, & Zandonella, 2010, p. 135).

These two case studies shed light on the fact that pro – and anti–asylum groups rely on unevenly distributed protest resources. Anti–asylum protesters represent the anger and grievances of voters. Pro–asylum supporters, instead, rely on altruistic convictions (Giugni & Passy, 2001). Pro–beneficiary actors, such as NGOs, charities and citizens, mobilize altruistically on behalf of individuals, with little or no institutional representation. This uneven political weight shapes the formation of protest–party linkages and their dynamics.

## Opportunistic versus substantive party activism

As the PEA data demonstrate, endogenous protest traits, such as moderate, implementation-targeted claims and the proximity of party actors to the grievances, combined with a lack of legal responsibility for the contentious issue, facilitate party activism. The in–depth cases reveal that the permeability of the protest arena is based on and causes diverging logics, asymmetric power configurations and unequal relationships between protest groups and political parties.

Below we synthesize the findings from both data sources and discuss the different shapes of party activism. We start with pro–asylum activism. When pro–asylum challenger and mainstream parties entered the protest arena, they transferred political weight and resources, e.g. public attention, to the protest; they aligned with protest groups on behalf of marginalized individuals. In general, actions by parties tended to be reactive and supportive rather than initiating protest events themselves. Activists continued to highlight the rights of migrants and place the focus on solidarity with ‘integrated’ residents, criticizing the perceived inhumane and unjust behaviour of the national government. Party competition was present but did not overshadow the issue of protecting the failed asylum seekers. The protest plotline mostly remained migrant–focused and did not shift towards competitive, political language. Rather, the protest repertoire was augmented through conventional parliamentary tools. The *anti–*asylum activism followed a different trajectory. Party activism tended to transform and reshape protests. Bottom–up activism was turned into top–down politics, which is also indicated by the comparatively high number of protest events in which political parties acted as initiators. The arena shifted from a space of substantive protest demands and goals towards opportunistic, competitive party politics.

Entering the protest arena is about not only communicating grievances and holding (other) decision makers accountable, as well as applying strategies to make these grievances heard and acted upon, it is also about distinguishing oneself from the decision makers, who are held responsible for these grievances (Jasper, 2015, p. 9). Party activism always contains some elements of party competition; however, the extent to which the protest is used as an episode of party competition and whether the nature of the protest will be transformed or not as a result can differ markedly. For pro–asylum protests, we find that party activism does not alter the substantive and bottom–up nature of the protests, whereas in the case of anti–asylum protests, party activism tends to be more opportunistic.

How can these differences between the pro – and anti–asylum protests be interpreted? We argue that the institutional opportunity of the presence in parliament of an anti–migration party enjoying high electoral success, which taps into and activates anti–migration attitudes, and the related discursive opportunities of a restrictive discourse around migration contribute to the different logics and extent of party activism for these protest types. Pro–asylum protests are organized against dominant perceptions and dominant political positions on migration. Political parties are willing to join these groups only if the public attitude and discourse in the specific case is favourable. In turn, anti–asylum protests are very much in sync with the dominant political positions and public attitudes on migration and asylum.

Considering the restrictive politicized environment, pro–asylum groups are particularly sensitive about the involvement of political parties. As the pro-asylum, family case demonstrates, given the politicized context, the activists tried to make a case around deservingness – this particular family/asylum seeker has earned the right to stay. This framing allowed them to gain the benevolence of the wider public and substantive support from political parties.

Challenging authorities in the case of facility protests is in line with the dominant public discourse and attitudes about migration. Party actors jump into the protest arena, often in an initiating role, knowing they have citizens and voters on their side. Party activism thus chimes with the public attitude. It mobilizes and acts upon, rather than challenges public opinion. In this sense, party activism in the field of migration can also be viewed as reflecting citizens’ preferences rather than ideological party lines. The Green Party goes partly against this trend, given that it largely refrains from anti–migration mobilization. Its position is to be interpreted as value–oriented. This disengagement and the party’s engagement in pro–migration protests is, however, in sync with the pro–migration preferences of their electorate (Dolezal, 2010, p. 544); hence it is also of electoral benefit to the party. As we noted before, there is usually an element of party politics at play, but the extent to which party politics transforms the protest arena is connected to whether or not the protests are in line with the dominant political position.

## Concluding summary

Previous scholarship has underlined growing party activism, and the fuzziness of the boundaries between the parliamentary and protest arenas. Against this background, this article dealt empirically with the endogenous and exogenous conditions of the protest arena to provide answers regarding party activism and how this engagement can transform the core features of the protest arena.

We proceeded in two steps. Based on a protest event analysis, we detected the degree of party activism in the asylum protest arena. Then we identified the major characteristics of those protest events that involved considerable party engagement. These protest–endogenous dimensions can be viewed as facilitating conditions for party activism in the protest arena. The second step was to study in–depth two key protest cases with the aim of revealing relevant political opportunities for the emergence of party–protest linkages. Besides, these case studies elucidate what kind of party activism modifies the purposes and goals of the protest arena and how it does so.

We conclude that political party actors, embedded in a political setting of overwhelmingly negative public opinions about the issues of asylum and migration, and a long established, electorally successful anti–migration party, are more prone to engage in anti-asylum than in pro-asylum protests. The greater involvement of parties in anti–asylum protests reflects this dominant negative political position towards the issue. In addition, however, parties also engage in pro-asylum protests. A condition that facilitates party activism, irrespective of whether protests are anti- or pro-asylum, is that the protest arena is characterized by modest, implementation–oriented claims. Political parties also turn to protests to challenge political authorities when they are not legally or politically responsible for the contentious decisions. The openness of the protest arena is, thus not related primarily to the type of party actor but to a party actor’s place in the decision–making process. Both challenger parties and those mainstream party organizations (on subnational levels) with no legal responsibility for the protest issue are active.

Our study indicates that party activism in the protest arena can occur for tactical, substantive or ideological reasons (Hutter, Kriesi & Lorenzini, 2018). We have shown that the consequences of party activism differ across protest arenas: parties are involved in the anti–asylum protest arena for competitive party politics ends. Party activism in pro–asylum protests, on the contrary, is grievance–based and parties support protesters without co–opting their goals. In line with this, pro–asylum protest actors express more sceptical views about the involvement of parties than protest groups that are sceptical about asylum, who are more open towards party actors.

The fact that parties, even in a country with a low level of protest culture, engage in the asylum protest arena at all confirms the protest scholarship that emphasizes the porosity of the boundaries between the streets and the parliament. However, the focus on Austria, the ‘heartland of the radical right’ (Gruber, 2014), also demonstrates that party activism in the arena of asylum protest is limited. This goes against previous findings on the extensive role of far–right parties in movements and protest activities (Caiani & Borri, 2016). Our findings underline that, in a setting with strong political parties, the politicization of migration remains largely within the confines of the parliamentary arena.

Further analysis of party–protest linkages would benefit from additional sources and research tools. The use of local media outlets and interviews with party actors would help to obtain a more complete picture of the permeable protest arenas. A cross–policy field comparison, including highly and less politicized areas, could reveal issue–related specifics and contribute to generally valid observations on the phenomenon. The same is true for cross–country comparisons, which would add insights regarding the political context sensitivities, in particular actor sensitivities in the protest arena. Since Austria attributes a very significant role to political parties within the political system, it would be fascinating to contrast our findings with different national frameworks in order to control for the overall role of parties and dominant cleavages.

## Note

1. The German keywords used were Flüchtlingslager, Flüchtlingsquartier, Flüchtlingsunterkunft, Asylheim, Asylzentrum, and Erstaufnahmezentrum, which are all synonyms for refugee accommodation, as well as the words Abschiebung (deportation) and abschieben (to deport).
